# Protein C deficiency in a uremic patient: a rare case of multivessel thromboembolism and type 5 cardiorenal syndrome

**DOI:** 10.1186/s12872-026-06085-0

**Published:** 2026-06-08

**Authors:** Qisu Ying, Jie Shen, Xiu Yang, Yu Wu, Hongtao Wu, Xuexue Shi, Sai Wu, Ning Zhao, Ming Wang, Xin Li, Yong Mao

**Affiliations:** 1 Department of Nephrology, Affiliated Hangzhou First People’s Hospital, School of Medicine, Westlake University, Hangzhou, Zhejiang 310006 China; 2 Department of Nephrology, Hangzhou First People’s Hospital Chengbei Campus, Hangzhou Geriatric Hospital, Hangzhou, Zhejiang 310022 China; 3 Department of Pharmacy, Affiliated Hangzhou First People’s Hospital, School of Medicine, Westlake University, Hangzhou, Zhejiang 310006 China

**Keywords:** Protein C deficiency, Type 5 cardiorenal syndrome, Heart failure, Renal failure, Case report

## Abstract

Protein C deficiency is a rare inherited disorder characterized by low levels or impaired function of protein C, a natural anticoagulant protein. Deficiency in protein C activity leads to a hypercoagulable state, predominantly resulting in venous thromboembolism. We report a case of a 69-year-old female with uremia who presented with recurrent heart failure, myocardial infarction, and extensive thromboembolism involving both arterial (coronary, abdominal aorta, renal, iliac, splenic, and femoral arteries) and venous systems (femoral, popliteal, and calf muscle veins). Progressive renal dysfunction led to a diagnosis of uremia, and genetic testing confirmed protein C deficiency. The patient ultimately succumbed to multiple organ failure, intractable ascites, and spontaneous bacterial peritonitis. This case underscores the importance of considering protein C deficiency in patients presenting with unexplained multivessel thromboembolism. For patients presenting with unexplained myocardial infarction, renal/hepatic dysfunction, extensive vascular thrombosis or calcification, acral gangrene, or persistently elevated D-dimer levels, we recommend implementing key diagnostic interventions, including protein C activity assay and genetic screening. This case suggests that protein C deficiency may be a rare potential etiology of type 5 cardiorenal syndrome, an association that requires further validation in larger cohort studies.

**Supplementary Information:**

The online version contains supplementary material available at 10.1186/s12872-026-06085-0.

## Introduction

Protein C, a liver-synthesized vitamin K-dependent anticoagulant zymogen, is activated by thrombin-thrombomodulin complexes to form active protein C (APC). APC inactivates coagulation factors V and VIII to suppress thrombosis. Reduced protein C activity induces hypercoagulability, increasing risks of thrombosis and disseminated intravascular coagulation (DIC), with advanced stages potentially causing coagulation dysfunction and bleeding [1]. It is linked to Budd-Chiari syndrome, arterial/venous thromboses, and in childhood hereditary cases, severe outcomes with purpura fulminans, blindness, or multi-organ microvascular thrombosis [2, 3]. However, several case reports have described adult patients with delayed disease onset despite having significantly decreased protein C activity [4, 5].

**Fig. 1:**
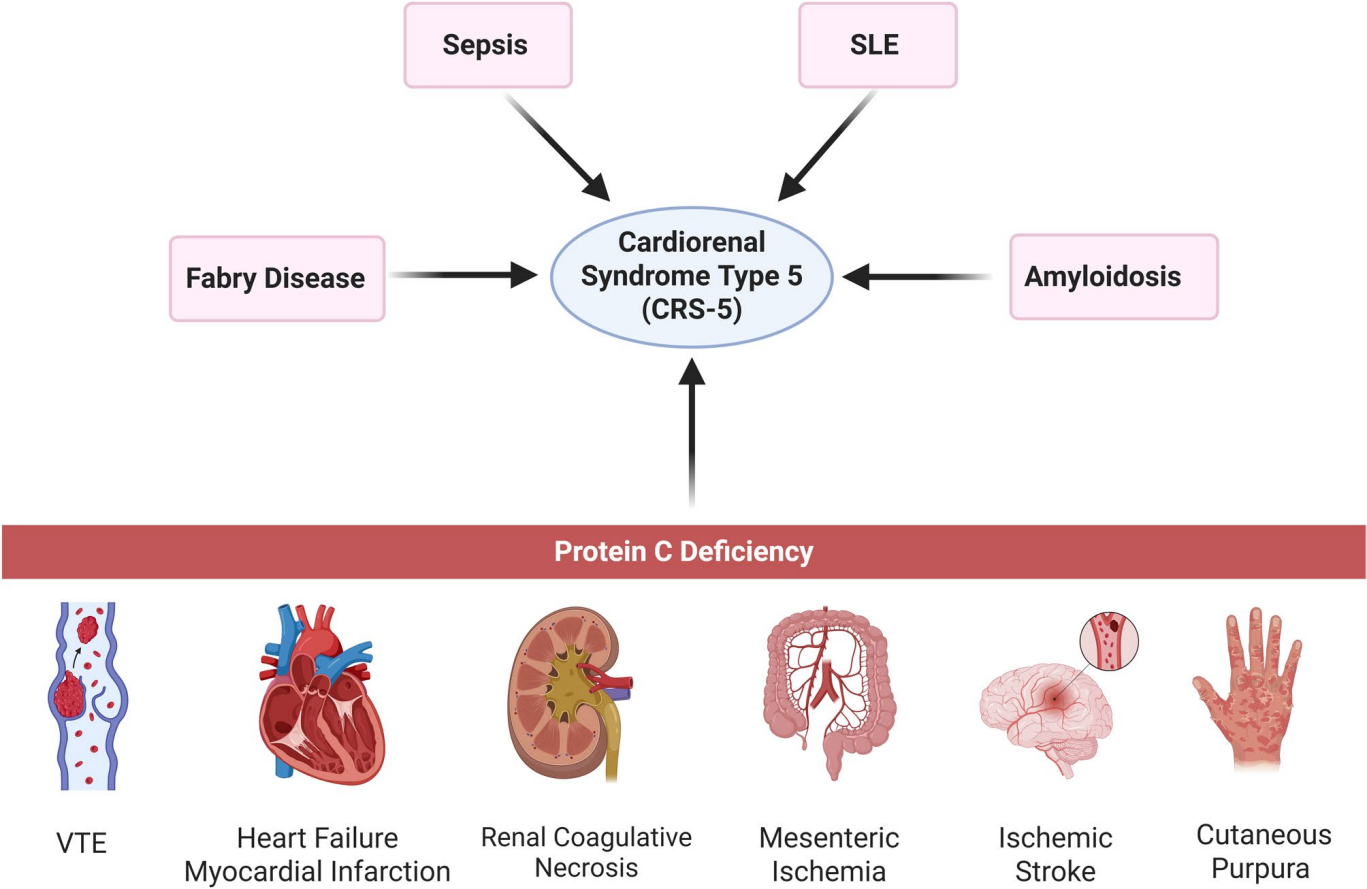
Protein C deficiency may constitute a rare etiology of type 5 cardiorenal syndrome. Figure created using BioRender.com. SLE, systemic lupus erythematosus; VTE, venous thromboembolism

Cardiorenal syndromes (CRS) involve disorders of heart or kidney whereby one organ dysfunction leads to dysfunction of another. Five types of CRS are defined. While first four types describe acute/chronic cardiorenal or renocardiac syndromes, type 5 CRS refers to secondary cardiorenal syndrome or cardiorenal involvement in systemic conditions and describes the concomitant presence of renal and cardiovascular dysfunction. Patients can develop type 5 cardiorenal syndrome in the setting of sepsis, systemic lupus erythematosus (SLE), Fabry disease, or amyloidosis; all of these disorders can lead to disease in both the heart and kidney (Fig. 1) [6]. 

This article reports a clinically critical case of a 69-year-old patient with late-onset protein C deficiency, characterized by predominant cardiorenal impairment and systemic multivascular thrombosis. The patient fulfilled the diagnostic criteria for type 5 cardiorenal syndrome. This case suggests a potential association between protein C deficiency and type 5 cardiorenal syndrome, and may provide preliminary insights into its possible pathogenetic mechanisms, requiring further verification in future studies.

## Case presentation

A 69-year-old female presented with a 3-year history of recurrent chest tightness and dyspnea, worsened by 2 weeks of anorexia. Over the past three years, she had multiple hospitalizations for acute myocardial infarction and coronary atherosclerotic heart disease. Percutaneous coronary intervention (PCI) of the left main-left anterior descending artery and right coronary artery initially improved her symptoms. Despite postoperative resolution of acute symptoms, she developed recurrent heart failure requiring repeated hospitalizations. Two weeks before admission, anorexia and abdominal distension emerged. Diagnostic workup revealed multi-serosal effusions (pleural, peritoneal, pelvic, pericardial), progressive hypoalbuminemia, and declining urine output. The patient had long-standing chronic comorbidities, including a 16-year history of hypertension with poor blood pressure control despite antihypertensive medication, over 10 years of type 2 diabetes mellitus poorly controlled by oral hypoglycemic agents combined with insulin therapy, and 9 years of dyslipidemia without regular lipid-lowering treatment. She was diagnosed with heart failure and acute-on-chronic renal failure, and transferred to nephrology for maintenance hemodialysis in August 2024. Auxiliary examinations conducted over the 3-year period since the onset of the patient’s condition are enumerated in Table 1.

**Table 1:** Timeline of auxiliary examination results

Year	Age	Symptoms	Evaluation	Main Diagnosis	Management
2021	66	chest tightness	ECG: Sinus rhythm with premature atrial contractions and ST-segment depression (0.03–0.06 mV in leads II, aVF, aVL, V4–V6). TTE: Left ventricular wall motion abnormality, left heart enlargement, left ventricular dysfunction (SimEF: 38%; E/e’: 36.3), pulmonary hypertension, minimal pericardial effusion, multiple atherosclerotic plaques in the aortic wall, and moderate mitral and tricuspid regurgitation. Chest CT: Cardiomegaly, pulmonary edema, pulmonary infection, pericardial and bilateral pleural effusion, with aortic and coronary atherosclerosis.	Coronary atherosclerotic heart disease Renal insufficiency	antiplatelet therapy ARNI therapy
2022	67	chest tightness dyspnea fatigue	Holter ECG: sinus rhythm, paroxysmal atrial fibrillation, and occasional ventricular premature beats. There is persistent ST-segment depression in lead V5 and intermittent ST-segment depression in lead aVF. Chest CT: pulmonary infection and edema, cardiac enlargement, pericardial effusion, and bilateral pleural effusion. Heart failure is suspected. Renal Ultra-Sound: urinary retention, accompanied by mild hydronephrosis in both kidneys and small renal cysts in the left kidney.	Coronary atherosclerotic heart disease Diabetic kidney disease? Heart failure Pulmonary edema Pneumonia	antiplatelet therapy diuretic therapy SGLT2 inhibitors ARNI therapy
2024	69	Chest tightness fatigue poor appetite	Speckle-tracking echocardiography: Left ventricular enlargement, diffuse left ventricular wall hypokinesia, increased left ventricular myocardial echogenicity, aortic valve local calcification, mild mitral and tricuspid valve regurgitation, SimEF 27%, small-volume pericardial effusion. Reduced left ventricular long- axis strain, preserved apical motion. Abdominal Vascular CTA: there were multiple atherosclerotic lesions in the abdominal aorta and its branches, severe stenosis in the initial segment of the left renal artery, severe stenosis in the left common iliac artery with penetrating ulcer, and mild to moderate stenosis in the remaining lumen. Abdominal CT: peritoneal effusion, splenomegaly, multiple calcifications in the spleen; possible calcification of blood vessel walls in both kidneys. Pathologic examination: No malignant cells were detected in ascites. No positive results were found in the abdominal wall fat biopsy and Congo red staining.	Protein C deficiency Renal failure Coronary atherosclerotic heart disease	Low molecular weight heparin Hemodialysis Drainage for ascites Human albumin treatment

*ECG* Electrocardiogram, *TTE* Transthoracic echocardiogram, *SimEF* Simulated Ejection Fraction, *CT* Computed Tomography, *CTA* CT angiography, *ARNI* Angiotensin Receptor-Neprilysin Inhibitor, *SGLT2* Sodium-dependent glucose transporters 2

The patient first presented with elevated serum creatinine (139 µmol/L) on February 4, 2021, and serum creatinine remained stable at 116–269 µmol/L during the following three years. On May 1, 2024, during admission for acute decompensated heart failure (NYHA class Ⅳ), her serum creatinine increased to 292 µmol/L. She was subsequently diagnosed with cardiorenal syndrome complicated by refractory volume overload and multiple serosal effusions. Hemodialysis was initiated on May 3, 2024. The dialysis regimen consisted of twice‑weekly hemodialysis plus once‑weekly hemodiafiltration, with each session lasting 2–3 h, and maintenance hemodialysis has been continued to date. Initial urinalysis was negative for protein, erythrocytes, and leukocytes, but subsequent tests showed small-to-moderate proteinuria. Urinary protein profiling showed total protein 16.2–37.7 mg/L, microalbumin 95.6–204.6 mg/L, IgG 8.02–53.2 mg/L, and N-acetyl-β-D-glucosaminidase (NAG) 2.8–10.2 U/L. Urine osmolality and specific gravity remained persistently low. Vasculitis antibodies, anti-phospholipase A2 receptor antibodies, autoantibodies, urinary light chains, and immunofixation electrophoresis were normal. Key abnormalities included hemoglobin 66–95 g/L, D-dimer 740–10,020 µg/L, ALT 30–83 U/L, AST 38–79 U/L, GGT 64–202 U/L, and ALP 167–474 U/L.

**Fig. 2:**
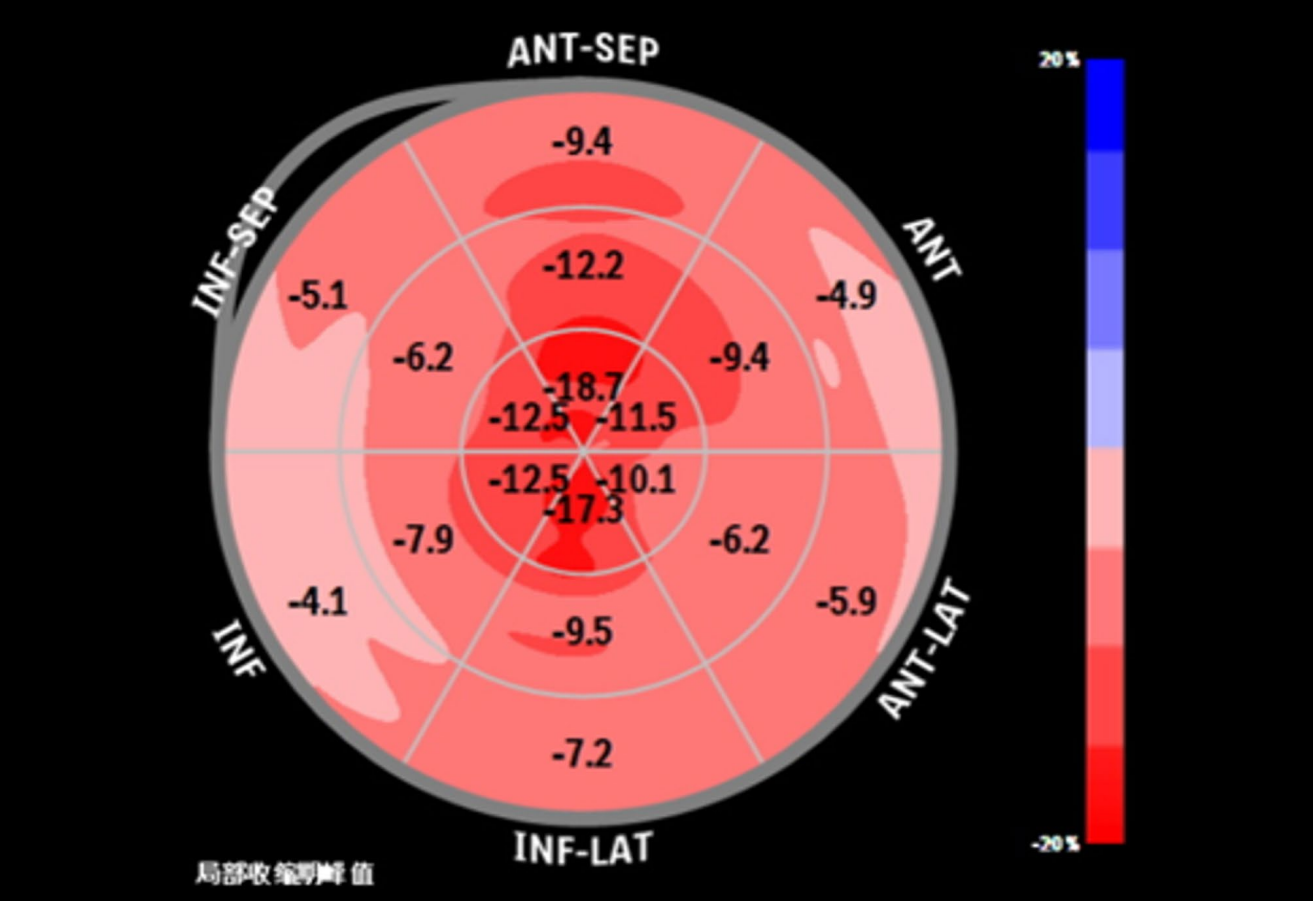
Speckle tracking echocardiography. Spect-tracking echocardiography indicated left ventricular enlargement, weakened left ventricular wall diffuse motion, enhanced left ventricular myocardial echo, local calcification of aortic valve, mild regurgitation of mitral and tricuspid valves, simEF: 27%, and a small amount of pericardial effusion

This female patient presented with insidious onset, prolonged course, and multisystem manifestations: renal/heart failure, serous effusions, anemia, splenomegaly, fatigue, and anorexia. Widespread systemic arteriovenous involvement was evident, with atherosclerosis, stenosis, and occlusion in coronary, abdominal aortic, left renal, left common iliac, splenic, and superficial femoral arteries, as well as superficial femoral, popliteal, and intramuscular veins—strongly indicating a systemic disease etiology. Cardiomegaly and heart failure were present. Three-dimensional ultrasound and speckle tracking echocardiography (Fig. 2) showed reduced left ventricular long-axis strain with preserved apical motion, raising suspicion of secondary cardiomyopathies (hypertensive cardiomyopathy, Fabry disease, amyloidosis).

Fabry disease typically manifests as left ventricular concentric hypertrophy with reduced inferoposterior wall longitudinal strain. Normal α-galactosidase A activity and globotriaosylceramide levels ruled out this diagnosis. Amyloid cardiomyopathy may present as recurrent heart failure, diastolic dysfunction, or abrupt blood pressure changes in hypertensive patients, with electrocardiographic findings like pseudo-infarction patterns or low QRS voltages. Normal blood/urine immunofixation electrophoresis, negative AL amyloidosis panel, wild-type transthyretin (TTR) gene sequencing, and negative abdominal fat biopsy (Congo red staining) excluded ATTR amyloidosis.

**Fig. 3:**
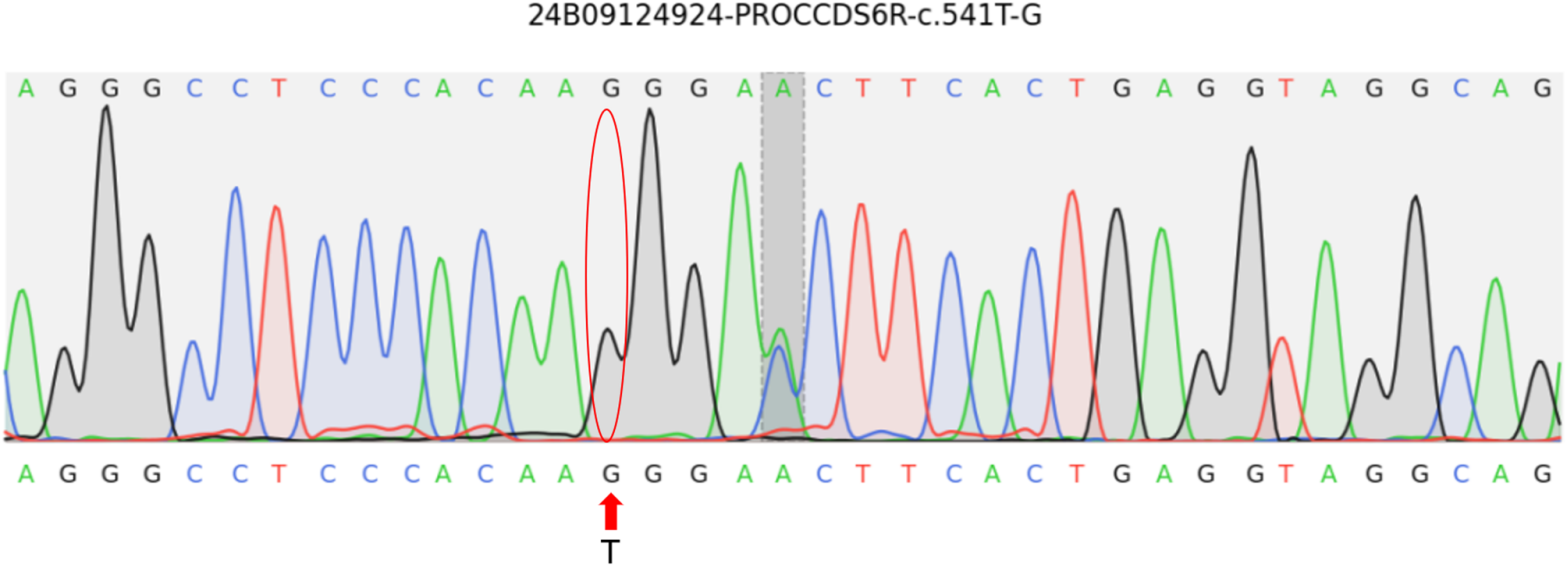
Sanger DNA sequencing validation of the PROC gene variant. The chromatogram shows the heterozygous c.541T > G substitution in the PROC gene (NM_000312.3). The red arrow marks the position of the variant, where both the wild-type thymine (T) and the variant guanine (G) allele are present, confirming the heterozygous genotype. This variant leads to an amino acid change (p.Phe181Val) that is associated with impaired protein C function

Whole-exome sequencing identified a heterozygous protein C gene mutation (c.541T > G) (Table 2), which was further confirmed by Sanger sequencing (Fig. 3). Table 2 summarizes the genetic variants identified by whole‑exome sequencing in the patient. The heterozygous PROC gene variant (c.541T > G; p.Phe181Val) is located in exon 7 (EX7/CDS6) and has been previously reported to be associated with reduced protein C activity, consistent with the patient’s clinical phenotype of protein C deficiency. The patient’s protein C activity of 47%, markedly below the normal reference range of 70%–140%, confirming partial protein C deficiency. This insufficient anticoagulant function predisposed the patient to a hypercoagulable state and acute thrombotic events rather than causing chronic atherosclerotic plaque formation.

**Table 2:** Validation site information

Gene	Transcript ID	Nucleotide variation	Amino acid variation	Gene subregion	Genotype	Chromosomal location
*PROC*	NM_000312.3	c.541T > G	p.Phe181Val	EX7/CDS6	Heterozygous	chr2:128183666–128183666

**Fig. 4:**
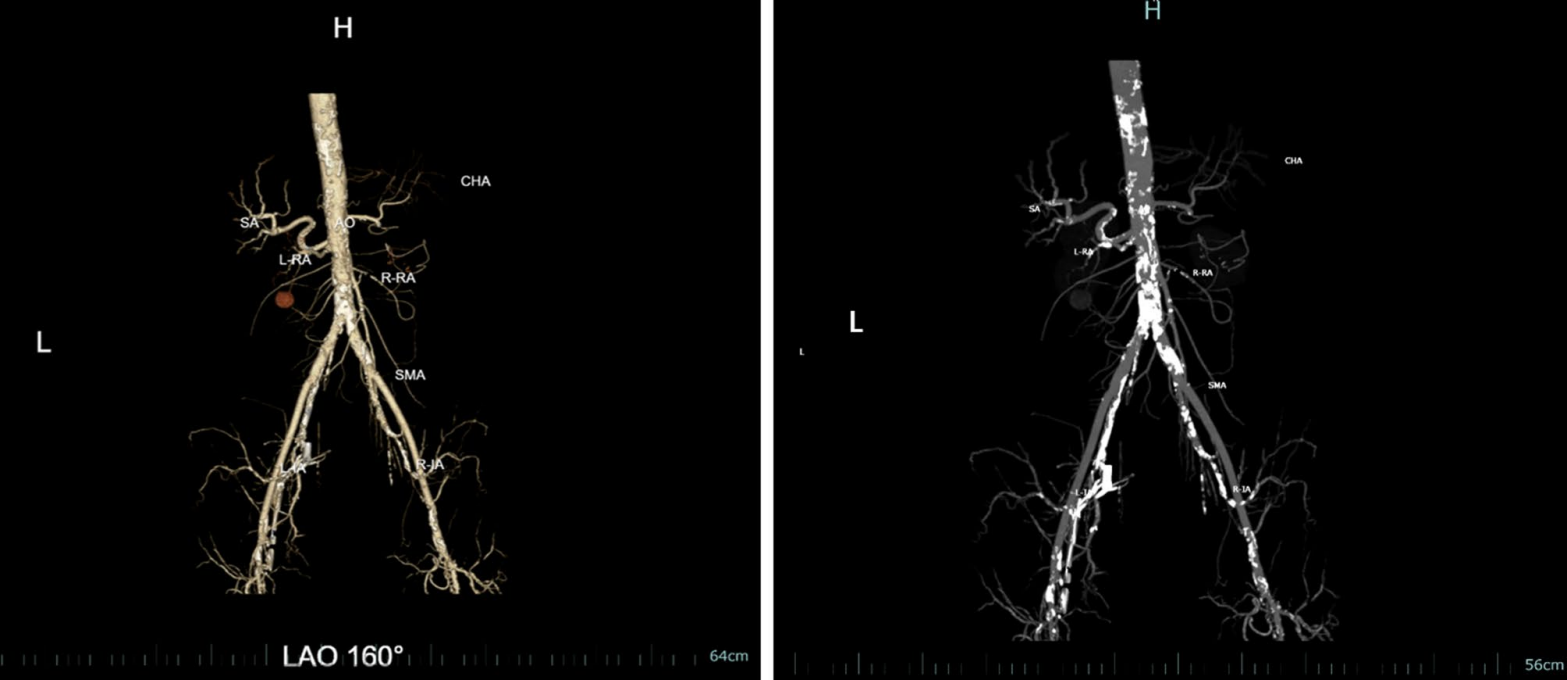
Abdominal vascular CTA showing extensive atherosclerotic lesions, arterial stenosis and penetrating ulcer of the abdominal aorta and its branches

To confirm the diagnosis, abdominal vascular CTA (Fig. 4) revealed multiple atherosclerotic lesions in the abdominal aorta and its branches, including severe stenosis at the origins of the left renal and left common iliac arteries. The latter was complicated by a penetrating ulcer, with mild-to-moderate stenosis in the remaining lumen.

**Fig. 5:**
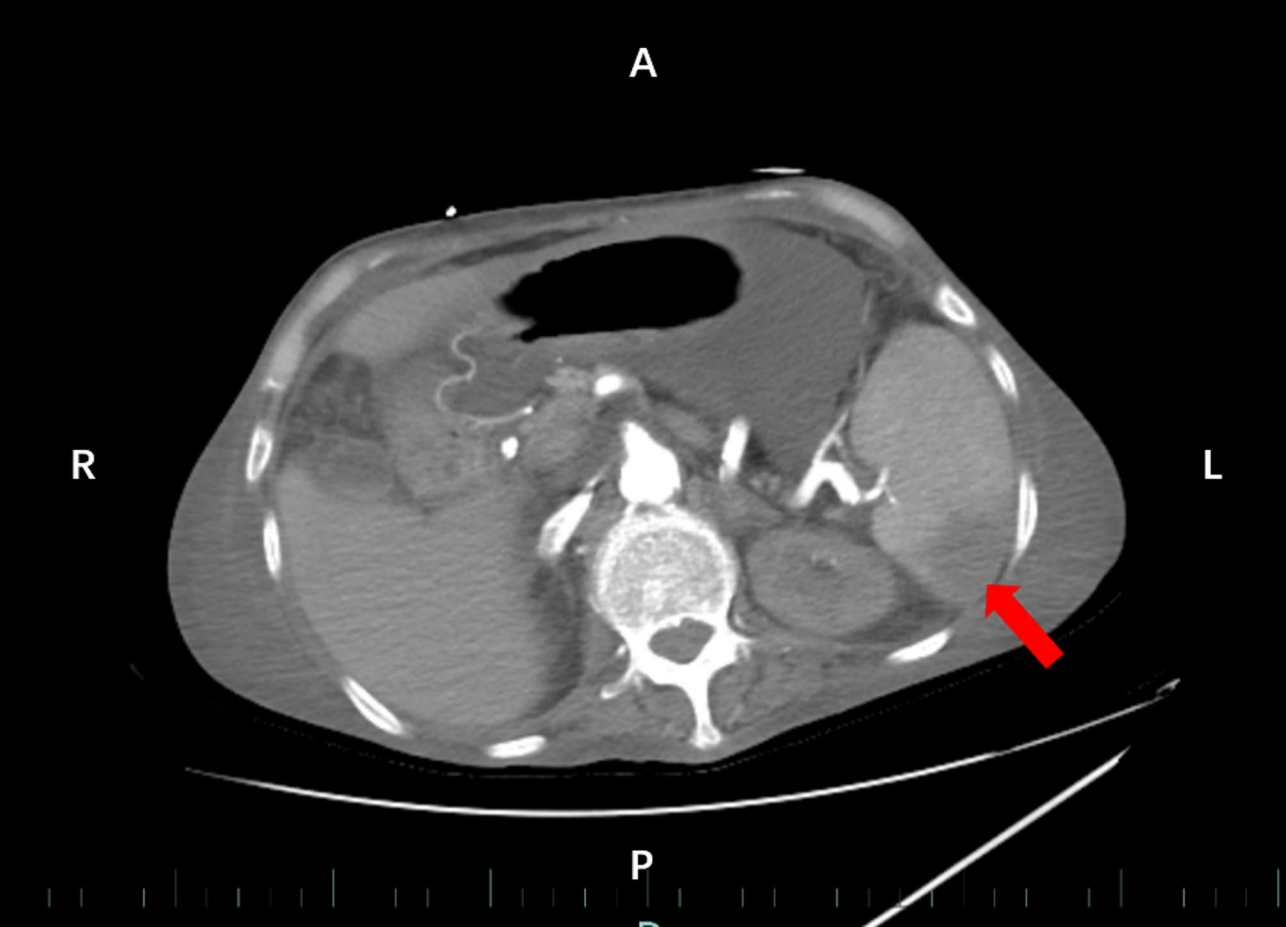
Abdominal CTA demonstrating splenic infarction and intestinal ischemic changes; the arrow indicates the infarcted area of the spleen

Extensive intestinal wall edema and thickening suggested ischemic changes, accompanied by abdominopelvic effusion and splenic infarction (Fig. 5). No signs of Budd-Chiari syndrome were found. Abdominal CTA showed widespread stenosis, occlusion, and calcification in the abdominal aorta and branches, involving the left renal, common iliac, splenic, and lower extremity vessels. The patient developed gangrene of the left first to fourth toes during hospitalization. We hypothesize a stepwise mechanism whereby protein C deficiency predisposed to cardiac and vascular thrombotic events, leading to progressive heart failure and secondary cardiorenal syndrome. Protein C deficiency may act as a key contributing factor rather than the primary cause of the underlying renal atherosclerotic lesions.

After diagnosing protein C deficiency, the patient was started on low-molecular-weight heparin for anticoagulation, and adjuvant activated protein C (APC) replacement was planned in accordance with current clinical evidence. After low-molecular-weight heparin administration, the patient’s D-dimer level remained persistently elevated, and systemic extensive thrombosis failed to be alleviated. During treatment, the patient developed serious complications including massive ascites, severe bacterial peritonitis, and hospital-acquired pneumonia, which greatly complicated anticoagulation management. Concerns about bleeding risk and severe infection made it difficult to maintain standardized long-term anticoagulation. The patient’s clinical condition continued to deteriorate, and she ultimately succumbed to progressive respiratory failure, leading to the discontinuation of all targeted anticoagulant treatments. Hemodialysis was administered to maintain uremia control, yet it failed to reverse the irreversible multiple organ dysfunction caused by combined infection and hypercoagulability.

## Discussion

Protein C deficiency is a recognized cause of arterial thrombosis (e.g., myocardial infarction, ischemic stroke). Mechanistically, it induces hypercoagulability via enhanced factor Xa/IXa activity, accelerating coronary atherosclerosis and myocardial infarction (MI) risk. In the present case, Long-standing hypertension and diabetes constituted the primary etiology of chronic atherosclerotic lesions and left renal artery stenosis observed in this patient. Protein C deficiency predisposes to acute thrombosis rather than chronic atherosclerotic plaque formation, but the hypercoagulable state may have exacerbated in-situ thrombosis on pre-existing atherosclerotic plaques and accelerated the clinical progression of vascular occlusion.

Previous case series and reports have consistently shown that hereditary protein C deficiency frequently presents with arterial thromboembolism, including acute myocardial infarction [7], multivessel cerebral arterial thrombosis [8], peripheral arterial occlusion [9], and mesenteric ischemia [10], often affecting young adults without significant traditional cardiovascular risk factors. Recent studies and case reports further confirm that protein C deficiency can cause widespread multivessel and multisystem arterial thrombotic involvement [11, 12], similar to the extensive arteriovenous thromboembolic phenotype observed in our patient. However, most previously reported cases presented at a young age and lacked severe underlying comorbidities such as long‑standing hypertension, diabetes, and uremia. In contrast, our patient was elderly and exhibited diffuse multivessel atherosclerosis combined with cardiorenal syndrome, representing a rare and distinct phenotype that expands the clinical spectrum of late‑onset, multiorgan‑involved protein C deficiency. A multi-institutional study [13] identified PROC gene variants as atherothrombosis risk factors, while Kario K et al. [14] first linked it to acute MI—a finding confirmed in young adults with protein C deficiency presenting with MI/stroke [10]. Conversely, higher protein C levels correlate with lower ischemic heart disease risk [15], underscoring its protective role and the need to assess protein C status in cardiovascular prevention.

Our patient was asymptomatic until her fifties, when she developed multivessel coronary occlusions, extensive arteriovenous thrombosis, and recurrent sepsis. This aligns with prior reports showing protein C deficiency can remain clinically silent until adulthood, typically presenting as myocardial infarction or severe thrombosis. For example, Grundy et al. [16] identified late-onset homozygous deficiency via DNA sequencing, and Tajima et al. [5] reported a 34-year-old with homozygous deficiency and thrombosis. A family study showed three heterozygous members developed MI or cerebral infarction at 50–55 years, while a recent study [17] confirmed protein C deficiency as an independent risk factor for pre-55 arterial thromboembolism, regardless of prior venous events.

A more pathophysiologically plausible mechanism is a stepwise cascade: protein C deficiency induced a hypercoagulable state, predisposing to coronary thrombosis, recurrent myocardial injury, and progressive heart failure [17, 18]. Severe cardiac dysfunction then led to secondary renal hypoperfusion, volume overload, and cardiorenal syndrome [19]. In this scenario, protein C deficiency serves as a critical contributing or secondary factor that precipitated clinical deterioration, rather than the primary cause of the underlying renal atherosclerotic stenosis [17]. The patient’s rapid progression to anuric renal failure prompted mechanistic analysis. Despite mild proteinuria, severe left renal artery stenosis—likely caused by protein C deficiency—was the primary pathology. Compounded by chronic peritoneal effusion, hypoproteinemia, and hypotension, renal perfusion was compromised, accelerating failure. Concurrent chronic heart failure (left ventricular ejection fraction 27–44% in 2021–2024) indicated type 5 cardiorenal syndrome (CRS), a secondary systemic disorder. While CRS-5 is typically associated with sepsis or cirrhosis [20], this case highlights a rare clinical phenotype of CRS-5 associated with protein C deficiency, providing useful clinical reference for similar cases.

Cardiorenal syndrome (CRS) encompasses disorders where acute/chronic dysfunction in one organ (heart/kidney) impairs the other, involving hemodynamic, neurohormonal, and inflammatory interactions [21]. Key mechanisms include RAAS overactivation, sympathetic hyperactivity, inflammatory cascades, and vasoactive substance imbalances [19, 22, 23]. Type 5 CRS (CRS-5) stands out for its systemic etiology and poor prognosis, often progressing to concurrent cardiorenal dysfunction with bidirectional injury: renal dysfunction drives cardiac remodeling, while cardiac impairment exacerbates renal hypoperfusion [19]. Standardized diagnostics and evidence-based therapies are crucial for CRS-5. Notably, protein C deficiency is more common in CKD—especially hemodialysis patients—with extracorporeal circulation exacerbating coagulation abnormalities and impacting survival [24], suggesting a role in CRS pathogenesis warranting further study.

Current evidence shows CRS-5 etiology extends beyond sepsis/diabetes to include systemic diseases like amyloidosis, SLE, and Fabry disease [19, 20]. Pathophysiologically, any systemic disease causing concurrent cardiorenal dysfunction can lead to CRS-5, but rare disease–related cases are often underdiagnosed, highlighting the need to explore diverse etiologies. To enhance diagnostic precision in cases of CRS-5 of uncertain etiology, a tailored, stepwise diagnostic protocol is recommended, commencing with basic screening for routine inflammatory and metabolic markers (e.g., CRP, HbA1c), followed by autoimmune evaluation via antibody profiling (e.g., ANA, anti-dsDNA), and concluding with specialized assays including serum free light chains, α-galactosidase A activity, and protein C quantification.

This patient demonstrated a rare and severe clinical course combining late‑onset thrombosis and sepsis, which carries important educational implications for clinical practice. Severe sepsis induces endothelial damage and systemic thrombosis while reducing endogenous APC levels via decreased hepatic production, thrombomodulin downregulation, and loss of endothelial protein C receptors [25], creating a vicious cycle that likely accelerated her thrombotic burden.

The association between sepsis and protein C deficiency remains poorly understood. Macias et al. [26] found severe protein C deficiency (< 40% of normal) in severe sepsis correlates with early mortality, where coagulation activation/inflammation severity dictates outcomes. Sequential protein C monitoring aids therapeutic assessment. Faust et al. [25] observed lower protein C levels, downregulated protein C receptor pathways, and endothelial dysfunction in meningococcal septicemia biopsies, implicating vascular endothelial protein C pathway dysfunction in sepsis-induced deficiency.

Current guidelines focus on pediatric purpura fulminans: 2018 ASH guidelines [27] recommend protein C concentrate (PC-C) or fresh frozen plasma (FFP) over anticoagulants for congenital cases. Virus-inactivated PC-C has been first-line for severe congenital deficiency since the 1990s, showing 94.7% response in purpura fulminans [28]. For late-onset adults, management is nuanced: novel oral anticoagulants (NOACs like dabigatran) avoid vitamin K antagonist–induced skin necrosis [29], while low-molecular-weight heparin (LMWH) alone or with PC-C may suffice for acute phases, as seen in a 24-month LMWH prophylaxis case for mild homozygous deficiency [30].

## Limitations

This report is a single-case retrospective study, which is inherently limited by its observational design. As a single case, it can only demonstrate an association rather than establish a causal relationship among uremia, decreased protein C activity, and the development of extensive arterial thrombosis and atherosclerosis. The individual contributions of underlying hypertension, diabetes, uremia-related inflammation, and protein C deficiency to the severe vascular phenotype cannot be quantitatively distinguished or validated. Furthermore, no long-term follow-up or family genetic screening was performed to further clarify the heritability and progression of this condition. Therefore, the conclusions should be interpreted with caution, and larger cohort studies are needed to verify the underlying mechanisms and causal links.

## Conclusions

In clinical practice, patients presenting with extensive thrombosis should undergo thorough evaluation of protein C levels. Genetic testing may be warranted to exclude protein C deficiency. Notably, protein C deficiency can rarely manifest as myocardial infarction or be triggered by sepsis. This case demonstrates that protein C deficiency may present as a rare etiology of cardiorenal syndrome. The value of this report lies in its rare clinical phenotype and educational significance for early recognition and multidisciplinary management. Adult-onset protein C deficiency is exceedingly rare, and large-scale clinical studies on its treatment are lacking, resulting in limited evidence-based guidance. Therefore, it is crucial to report more cases, share clinical experiences, and summarize treatment strategies to advance understanding and management.

## Supplementary Information

Supplementary Material 1.

## Data Availability

The data used to support the findings of this study are included within the article.

## Abbreviations

APC: Activated protein C
DIC: Disseminated intravascular coagulation
CRS: Cardiorenal syndrome
CRS-5: Type 5 cardiorenal syndrome
SLE: Systemic lupus erythematosus
VTE: Venous thromboembolism
PCI: Percutaneous coronary intervention
ECG: Electrocardiogram
TTE: Transthoracic echocardiogram
SimEF: Simulated ejection fraction
CT: Computed tomography
CTA: CT angiography
ARNI: Angiotensin receptor-neprilysin inhibitor
SGLT2: Sodium-dependent glucose transporters 2
NYHA class Ⅳ: New York Heart Association functional class IV
NAG: N-acetyl-β-D-glucosaminidase
ALT: Alanine transaminase
AST: Aspartate transaminase
GGT: Gamma-glutamyl transferase
ALP: Alkaline phosphatase
AL: Amyloid light-chain
TTR: Transthyretin
ATTR: Amyloid transthyretin
DNA: Deoxyribonucleic acid
MI: Myocardial infarction
RAAS: Renin-angiotensin-aldosterone system
CKD: Chronic kidney disease
CRP: C-reactive protein
ANA: Antinuclear antibodies
anti-dsDNA: Anti-double-stranded DNA
ASH: American Society of Hematology
PC-C: Protein C concentrate
FFP: Fresh frozen plasma
NOACs: Novel oral anticoagulants
LMWH: Low-molecular-weight heparin
